# Presenilin1 exerts antiproliferative effects by repressing the Wnt/β-catenin pathway in glioblastoma

**DOI:** 10.1186/s12964-019-0501-9

**Published:** 2020-02-11

**Authors:** Wei Yang, Peng-fei Wu, Jian-xing Ma, Mao-jun Liao, Lun-shan Xu, Min-hui Xu, Liang Yi

**Affiliations:** Department of Neurosurgery, Daping Hospital & Institute Research of Surgery of Army Medical University, 10# Changjiangzhi Road, Daping, Yuzhong District, Chongqing, 400042 China

**Keywords:** Glioblastoma, Presenilin1, Proliferation, Cell cycle

## Abstract

**Background:**

Glioblastoma and Alzheimer’s disease (AD) are the most common and devastating diseases in the central nervous system. The dysfunction of Presenilin1 is the main reason for AD pathogenesis. However, the molecular function of Presenilin1 and its relative mechanism in glioblastoma remain unclear.

**Methods:**

Expression of presenilin1 in glioma was determined by IHC. CCK-8, colony formation, Flow cytometry, Edu staining were utilized to evaluate functions of presenilin1 on glioblastoma proliferation. The mechanism of above process was assessed by Western blotting and cell immunofluorescence. Mouse transplanting glioblastoma model and micro-MRI detection were used to verified presenilin1 function in vivo.

**Results:**

In this study, we found that all grades of glioma maintained relatively low Presenilin1 expression and that the expression of Presenilin1 in high-grade glioma was significantly lower than that in low-grade glioma. Moreover, the Presenilin1 level had a positive correlation with glioma and glioblastoma patient prognosis. Next, we determined that Presenilin1 inhibited the growth and proliferation of glioblastoma cells by downregulating CDK6, C-myc and Cyclin D1 to arrest the cell cycle at the G1/S phase. Mechanistically, Presenilin1 promoted the direct phosphorylation of β-catenin at the 45 site and indirect phosphorylation at the 33/37/41 site, then decreased the stabilized part of β-catenin and hindered its translocation from the cytoplasm to the nucleus. Furthermore, we found that Presenilin1 downregulation clearly accelerated the growth of subcutaneous glioblastoma, and Presenilin1 overexpression significantly repressed the subcutaneous and intracranial transplantation of glioblastoma by hindering β-catenin-dependent cell proliferation.

**Conclusion:**

Our data implicate the antiproliferative effect of Presenilin1 in glioblastoma by suppressing Wnt/β-catenin signaling, which may provide a novel therapeutic agent for glioblastoma.

Video Abstract.

## Backgroud

Glioma is the most frequent and lethal brain tumor in adults, accounting for 45–50% of all primary intrinsic brain tumors and 75% of all malignant tumors in the central nervous system. Glioblastoma multiforme (GBM), the highest grade of glioma, is characterized histologically by considerable cellularity and mitotic activity, vascular proliferation and necrosis [1]. The current management strategy of glioblastoma includes surgery followed by radiotherapy and chemotherapy with temozolomide [2]. Despite the development of therapeutic methods, the median survival time of glioblastoma is still approximately 14.5 months and only 5.5% of patients survive up to 5 years [3]. Thus, identifying the molecules that regulate GBM progression and accurate molecular mechanisms may contribute to the exploration of reliable therapeutic targets for glioblastoma.

Current estimates suggest that approximately 44 million people live with dementia worldwide, and among those patients, Alzheimer’s disease (AD) is the single major cause of dementia, accounting for 50–75% [4]. However, glioma is barely found in AD patients. This characteristic suggests that there may be an antiglioma molecule in AD pathogenesis. Presenilin1 is encoded by the PS1 gene, which is located on chromosome 14 and contains 467 amino acids in humans [5]. Presenilin1 is a catalytic component of the γ-secretase complex responsible for the intramembranous cleavage of AD-related amyloid precursor protein (APP). The dysfunction of the Presenilin1 gene leading to aberrant APP cleavage is central to AD pathogenesis [6]. Presenilin1 was first found in familial autosomal dominant AD in 1995 [7]; Roperch, JP. reported that the downregulation of Presenilin1 in lymphatic sarcoma U937 cells results in reduced growth and increased cell apoptosis [8]. Moreover, Presenilin1 regulates the paired phosphorylation of β-Catenin independently of Axin/CK-1α in tumorigenesis [9]. Presenilin1 was reported to be involved in ovarian cancer [10], gastric cancer [11], bladder cancer [12], colorectal cancer [13] and hepatocellular carcinoma [14]. However, despite AD being most frequent disease of neurology and glioblastoma being most common tumor of neurosurgery departments, the detailed roles of this AD-related molecule in glioblastoma have not yet been thoroughly validated, and the underlying mechanism needs to be clarified.

In this study, we investigated the expression levels of Presenilin1 in human glioma tissues. We analyzed the correlation between Presenilin1 expression and the prognosis of patients with glioma. We used gene silencing and overexpression to investigate the effects of Presenilin1 on glioblastoma proliferation. We found that Presenilin1 regulated glioblastoma mainly through Wnt/β-catenin signaling in vitro and in vivo. Our results suggest that Presenilin1 prevents glioblastoma progression and may be a novel prognostic factor for glioblastoma. Importantly, the stimulation of Presenilin1 could be developed as a promising therapeutic method for human glioma patients.

## Materials and methods

### Cell culture

Human glioblastoma cell lines (U87 and U251) were purchased from the American Type Culture Collection (ATCC;USA) and were cultured in DMEM/F12(Hyclone, USA) supplemented with 10% fetal bovine serum (FBS; Gibco), 1% penicillin and streptomycin (Beyotime). All the cells were cultured in a humidified incubator at 37 °C with 5% CO2.

### Reagents and antibodies

DMSO and DAPI were obtained from Sigma Aldrich. Lipofectamine 2000 was obtained from Invitrogen. Rabbit anti-Presenilin1 monoclonal antibody was purchased from Proteintech (Wuhan, China). Anti-GSK-3β, p-GSK-3β, and Actin antibodies were purchased from Beyotime, β-Catenin Antibody Sampler Kit, C-myc, cyclinD1 and CDK6 antibodies were purchased from CST.

### Analysis of patient data

The data was downloaded from TCGA database on April 3, 2018 (https://portal.gdc.cancer.gov). After deleted the uncertainty data and lacking of clinical data, the RNA-seq data contained 272 glioma patients information. Kaplan–Meier analysis was conducted with the TCGA-glioma, TCGA-gliomblastoma and TCGA-low grade glioma datasets. we used cutoff value at 4854 to divide211 glioma patients and cutoff value at 4794to divide 119 glioblastoma patients into two groups (high expression and low expression) for analyze overall survival (OS) rates. we used cutoff value at 3722 to divide 272 glioma patients into two groups (high expression and low expression) for analyze recurrence rates, we failed to found appropriate cutoff value to divide low grade glioma and make significantly difference between two groups. The OS and RFS curve and *P* value were analyzed by log-rank test.

### Immunohistochemistry (IHC)

Human glioma tissues were fixed and cut into 5 μ m sections. The slides were treated for antigen retrieval. Sections were incubated with primary antibody (1:50) for overnight at 4 °C, then were visualized using a DAB detection kit according to the manufacturer’ s protocol. Sections were counterstained with hematoxylin and observed under a BX-53 microscope (Olympus, Tokyo, Japan). The degree of immunostaining in the sections was reviewed and scored independently by 2 observers based on both the percentage of positive-stained tumor cells and the staining intensity. The staining intensity was graded in four categories on a scale from 0 to 3 (intensity scores): no staining (0), light-brown staining (1), brown staining (2) and dark-brown staining (3). Protein staining was evaluated using the following formula: overall staining score = intensity score×percentage score.

### Lentivirus transfection

To generate stably over expression cells, U87 and U251 cells were transfected with lentiviral containing with Presenilin1 sequence (Lv-PS1) or with empty vector (Lv-Ctr), which were constructed by Genechem (Shanghai, China), and U87 and U251 cells were transfected with lentiviral containing the sh-Presenilin1 sequence (sh-PS1) or with the negative control vector (sh-Ctr), which were constructed by Genechem (Shanghai, China). Cells were incubated in medium containing the virus and polybrene (5 μg/ml) for 48 h. After infection, cells were selected by culture with puromycin concentration at 4 μg/ml for 3 days and at 1 μg/ml for 2 weeks.

### CCK-8 assay

Transfected glioma cells were seeded in 96-well plates at 1 × 10^3^ every well. After the cells were adherent, the culture medium was replaced. The CCK8 (Dojindo Laboratories, Japan) assay was then conducted according to the manufacturer’s instructions, and the absorbance at a wavelength of 450 nm was read using a microplate reader every day at the same time for 5 days.

### Colony formation assay

Transfected glioma cells were seeded in 3 cm dish plates (800 cells/well). After cultured at 37 °C for 14 days, colonies were formed. Then the cells were fixed with 4% paraformaldehyde for 30 min and stained with crystal violet solution for 10 min. The number of colonies (> 50 cells/colony) was quantified using a microscopy.

### Cell cycle assay

Cell cycle progression was examined on a flow cytometer using propidium iodide (PI) staining. Cells infected with lentivirus against or over-expressed of Presenilin1gene. Then cells were seeded in six-well culture plates and cultured to 80% confluence. Cell cycle was analyzed by PI staining of nuclei. PI absorbance was determined by fluorescence activated cell sorting on a flow cytometry (ACEA, San Diego, USA).

### EdU staining assay

2 × 10^4^ transfected glioma cells were seeded into con-focal dish and adherent, and fixed with 4% paraformaldehyde, then were treated with 2 mg/ml glycine and permeated with 0.5% TritonX-100, the EdU staining was preformed according to the manufacturer’s procedure of Cell-Light EdU apollo567 in vitro kit (Rui-bio, Guangzhou, China), cells were counter-stained with DAPI. The dishes were observed with confocal microscopy.

### Western blotting assay

The total protein was extracted using RIPA lysis buffer (Beyotime, Shanghai, China) with 1% PMSF (Beyotime) and 1x Phosphorylase inhibitors (Solabio, Beijing, China), and BCA kit (Beyotime) was used to measure the concentration according to the manufacturer’s instructions. The proteins were separated by SDS-PAGE gel electrophoresis and transferred to PVDF membrane (Millipore). The membrane was blocked and incubated with primary antibodies (Presenilin1,1:2000; GSK-3β, 1:2500; p9-GSK-3β, 1:2500; β-Catenin,1:2000; p45-β-Catenin,1:2000; p33/37/41-β-Catenin,1:2000; Actin,1:5000) and the IgG-hydrogen peroxide (HRP) secondary antibody in sequence. The protein blots were exposed to Clarity™ Western ECL substrate (BIO-RAD) using the BIO-RAD ChemiDoc™ Touch Imaging System.

### Immunofluorescence

Cells grown on co-focal dish were fixed with 4% paraformaldehyde, permeated and blocked. Next, the cells were incubated with the primary antibody (β-Catenin,1:100) at 4 °C overnight followed by TRITC-conjugated goat anti-rabbit IgG (1:200). Dish were counterstained with DAPI to visualize cell nuclei. Images were recorded using a co-focal laser scanning microscope (Leica TCS SP8, Germany) The colocalization was analyzed with Image J software.

### Tumor xenografts

All experimental procedures were approved by the Institutional Animal Care and Use Committee of army Medical University. Four-week-old mice were randomly divided into two groups (*n* = 5 per group), and implanted with 1 × 10^6^ cells previously transfected with Presenilin1 shRNA lentivirus or Presenilin1overexpressing lentivirus, as well as negative control respective. Cells were mixed with Matrigel (50% volume) and implanted subcutaneously into the inguinal folds of the nude mice. Tumor volume was determined using an external caliper and calculated by using the Formula = (Length×Width^2^)/2. Mice were sacrificed 21 days after transplantion. At this point, tumors were excised and subjected to downstream analyses. For the orthotropic model, 1 × 10^5^ U87-Lv-Ctr and U87-Lv-PS1 cells were injected into the right corpus striatum of the brains of 6 week-old nude mice using a stereotactic frame. Micro-MRI(7.0 T, Bruker BioSpin MRI GmbH, Germany) was performed to record the growth of tumor at 7 days and 19 days after injection. The tumor values calculated by using the Formula = (π/6) × length×width×depth. Kaplan-Maier survival curves were calculated using GraphPad Prism 6.0 software. Mice were monitored and sacrificed when neurological signs appeared or after 40 days.

### Statistical analysis

All experiments were performed in triplicate. Statistical analyses were carried out by using SPSS 13.0 statistical software. Data were exhibited as means ± SD. Statistical significance was calculated by Student’s t-test or one-way ANOVA. Survival was analyzed by Kaplan-Meier method and compared by log-rank test by GraphPad Prism 5. (*) *p* < 0.05 was considered statistically significant.

## Results

### Presenilin1 is downregulated in malignant glioma and is positively correlated with the prognosis of glioma patients

**Fig. 1:**
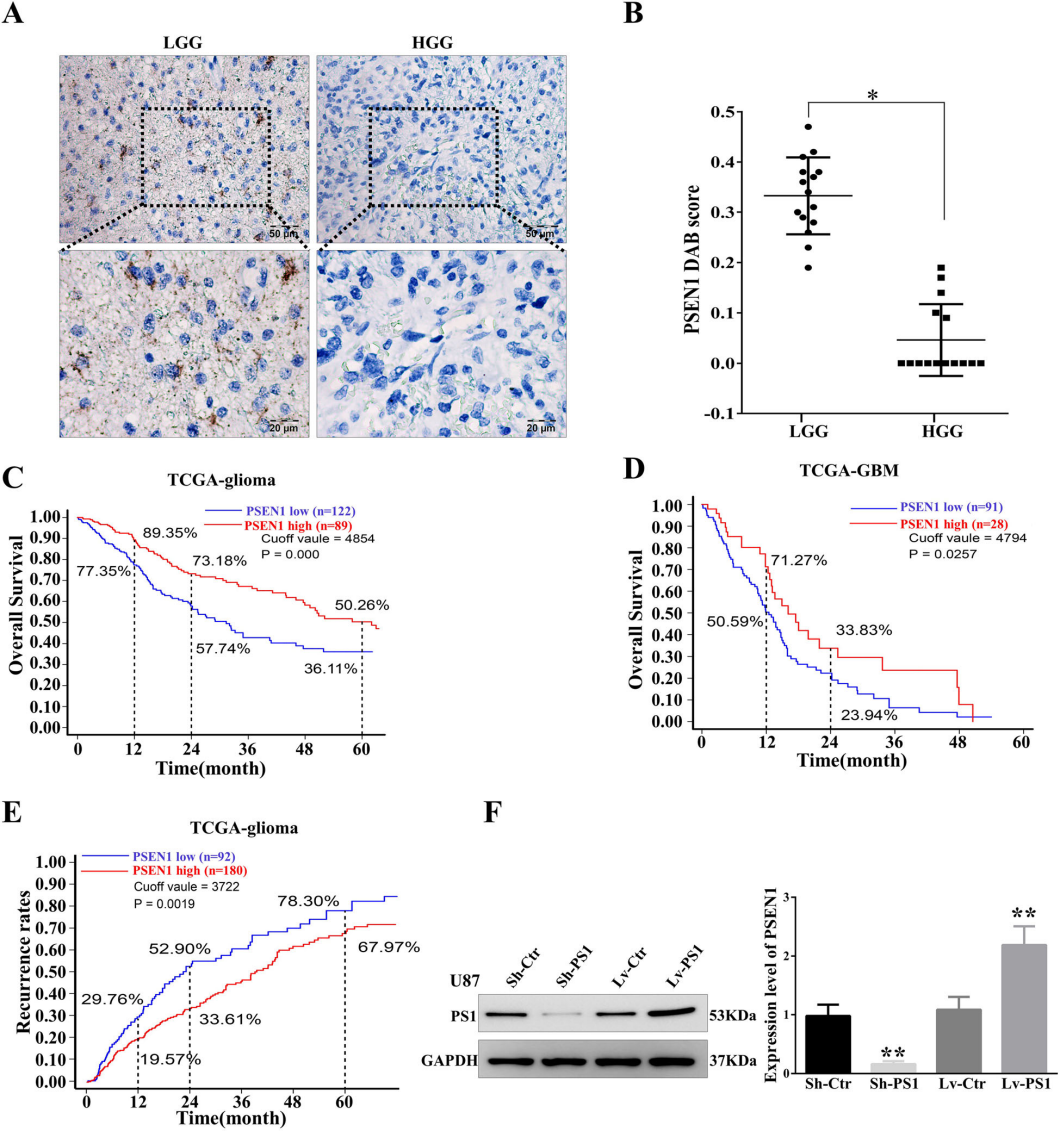
**a** Representative IHC staining of Presenilin1 protein in low grade glioma tissues (LGG, left panels) and high grade glioma tissues (HGG, right panels). Scale bars = 50 um and 20 um respectively. **b** Statistical analysis of the relative expression levels of Presenilin1 in LGG and HGG, * *p* < 0.05. **c** Kaplan-Meier analysis for all grade glioma patients, patients in the high Presenilin1 group (*n* = 122) and in the low Presenilin1 group (*n* = 89) (*P* = 0.0000, log-rank test). **d** Kaplan-Meier analysis for glioma patients. Patients in the high Presenilin1 group (*n* = 28) and in the low Presenilin1 group (*n* = 91) (*P* = 0.0257, log-rank test). **e** Kaplan-Meier analysis for all grade glioma patients. Patients in the high Presenilin1 group (*n* = 180) and in the low Presenilin1 group (*n* = 92) (*P* = 0.0019, log-rank test). **f** U87 cells were transfected with lentivirus contain sh-RNA target Presenilin1(sh-PS1) or full-length gene of Presenilin1(Lv-PS1).Sh-NC and Lv-NC were used as control, then Western-blot to detect the down- and over-expression effect of lentivirus in U87 cells, *p < 0.05,**p < 0.01

To explore the potential connection between Presenilin1 and human glioma, we first performed immunohistochemistry (IHC) to detect the expression of Presenilin1 in glioma tissues. The IHC results showed that Presenilin1 was primarily located in the cytoplasm and membrane, and Presenilin1 was slightly stained in low-grade glioma (LGG). However, Presenilin1 was barely expressed in high-grade glioma (HGG) (Fig. 1a). Interestingly, we found that presenilin1 was especially overexpressed in the pseudopalisading necrosis area of human glioblastoma tissues (data not shown). Semiquantitative analysis of Presenilin1 showed that the DAB scores of LGG were significantly higher those of HGG (Fig. 1b). Next, we performed Kaplan-Meier analysis to investigate the relationship between Presenilin1 expression and the overall survival (OS) rates of glioma patients. In all grades of glioma patients, using a cutoff value to divide 183 patients into a Presenilin1-high (Presenilin1^high^) group and a Presenilin1-low (Presenilin1^low^) group, we found that the 1-year, 2-year and 5-year OS rates of patients in the Presenilin1^high^ group were significantly higher than those in the Presenilin1^low^ group (Fig. 1c). Similarly, in GBM patients, the 1-year and 2-year OS rates of patients in the Presenilin1^high^ group were significantly higher than those of patients in the Presenilin1^low^ group (Fig. 1d). However, we failed to find an appropriate cutoff value to divide LGG patients into high and low groups, which had significantly different OS rates (Additional file 1: Figure S1A). We also utilized Kaplan-Meier analysis to observe the correlation of Presenilin1 expression with the recurrence rates of glioma patients and found that the 1-year, 2-year and 5-year recurrence rates in the Presenilin1^high^ group were significantly lower than those in the Presenilin1^low^ group (Fig. 1e). However, Presenilin1 could not be a prognostic marker for glioblastoma or LGG patients (data not shown). The above results suggested that the Presenilin1 level was positively correlated with the prognosis of glioma and GBM patients.

We found that Presenilin1 was also expressed at low levels in U87 and U251 cell lines, so we accumulated approximately 10-fold more cells than the general protocol to extract whole cell protein for western blot detection. In addition, we used lentivirus infection to upregulate and downregulate Presenilin1 in U87 and U251 cells. The western blotting results showed that the expression of Presenilin1 protein was decreased approximately 90% by Sh-PS1 and increased 1.5-fold by Lv-PS1 compared to the control (Fig. 1f & Additional file 1: Figure S1B).

### Presenilin1 represses the growth of glioblastoma cells in vitro

**Fig. 2:**
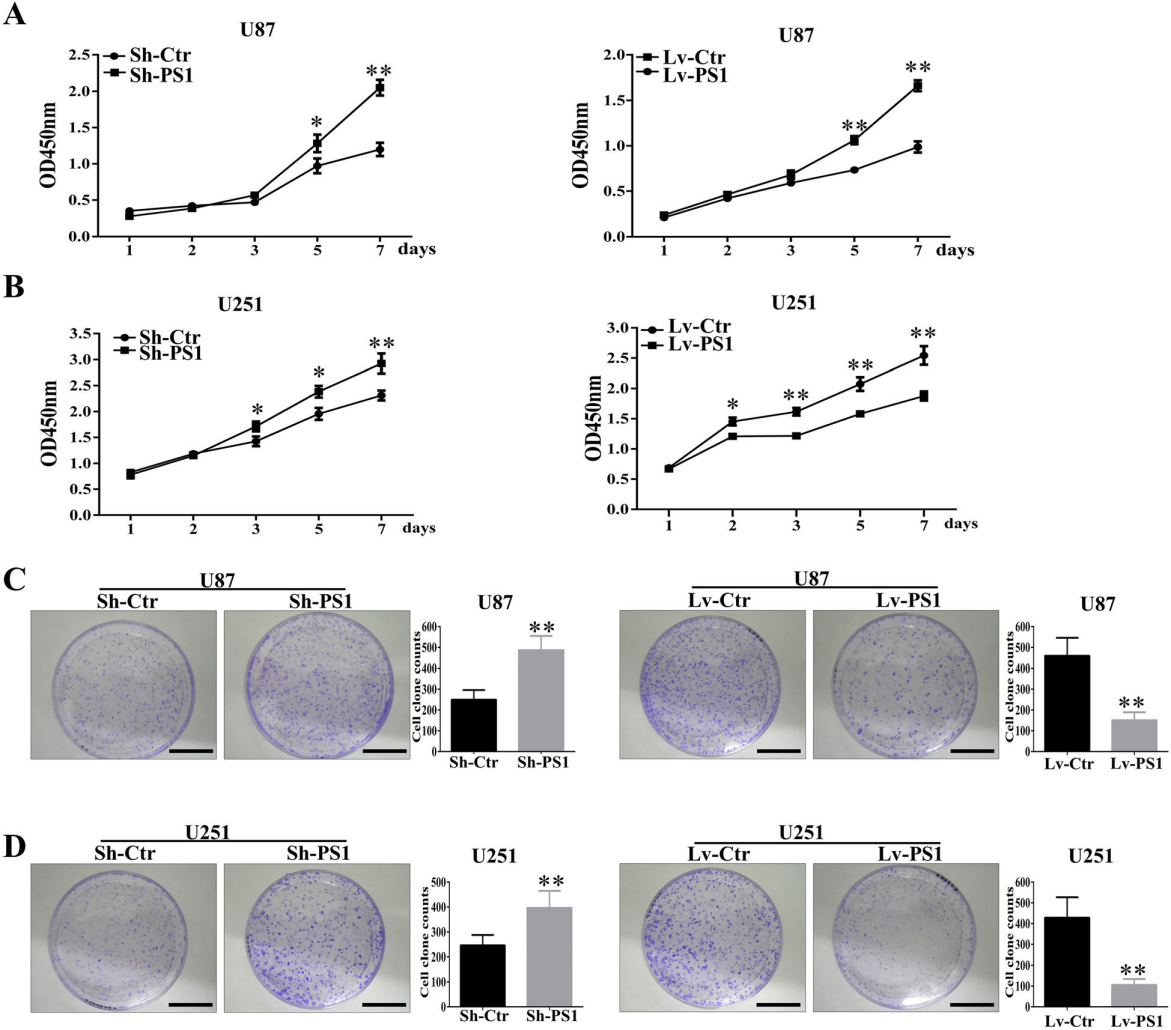
**a** CCK-8 assays were performed in U87 cell when loss- or gain- function of Presenilin1, OD value were detected at day 1,2,3,5 and 7 and were showed as mean ± SD. **b** U251 cells down- or up-expressed of Presenilin1, then were used for CCK-8 assays to reveal the function of Presenilin1 in U251 cells. **c** Clone formation of the U87 cells after knockdown or over-expression of Presenilin1, histograms showing the clone formation in each group, Scale bars = 1 cm. **d** Representative image of clone formation in U251 cells after Sh-Presenilin1 or Lv-Presenilin1 treatment, Scale bars = 1 cm.* *p* < 0.05,**p < 0.01

The positive correlation between Presenilin1 and glioma prognosis led us to explore the antitumor function of Presenilin1 in glioblastoma. First, we investigated the role of Presenilin1 in glioma cell growth by CCK-8 assay. As shown in Fig. 2a, Presenilin1 depletion significantly promoted the growth ratio of U87 cells, and the upregulation of Presenilin1 significantly inhibited U87 cell growth. Likewise, the downregulation of Presenilin1 accelerated cell growth, and the upregulation of Presenilin1 decelerated the growth of U251 cells (Fig. 2b). Simultaneously, we performed colony formation assays to detect the effect of Presenilin1 on glioma. The colony number of U87 cells significantly increased when sh-PS1 was transfected, and this number sharply decreased when Lv-PS1 was transfected (Fig. 2c). Similarly, we observed that Presenilin1 negatively regulated the colony formation ability of U251 cells (Fig. 2d). Thus, the above results illustrated that Presenilin1 hindered the cell growth of glioblastoma cells in vitro*,* which could partly explain the positive relation between Presenilin1 and the prognosis of glioblastoma.

### Presenilin1 inhibits glioblastoma proliferation by arresting the G1/S phase of the cell cycle in vitro

**Fig. 3:**
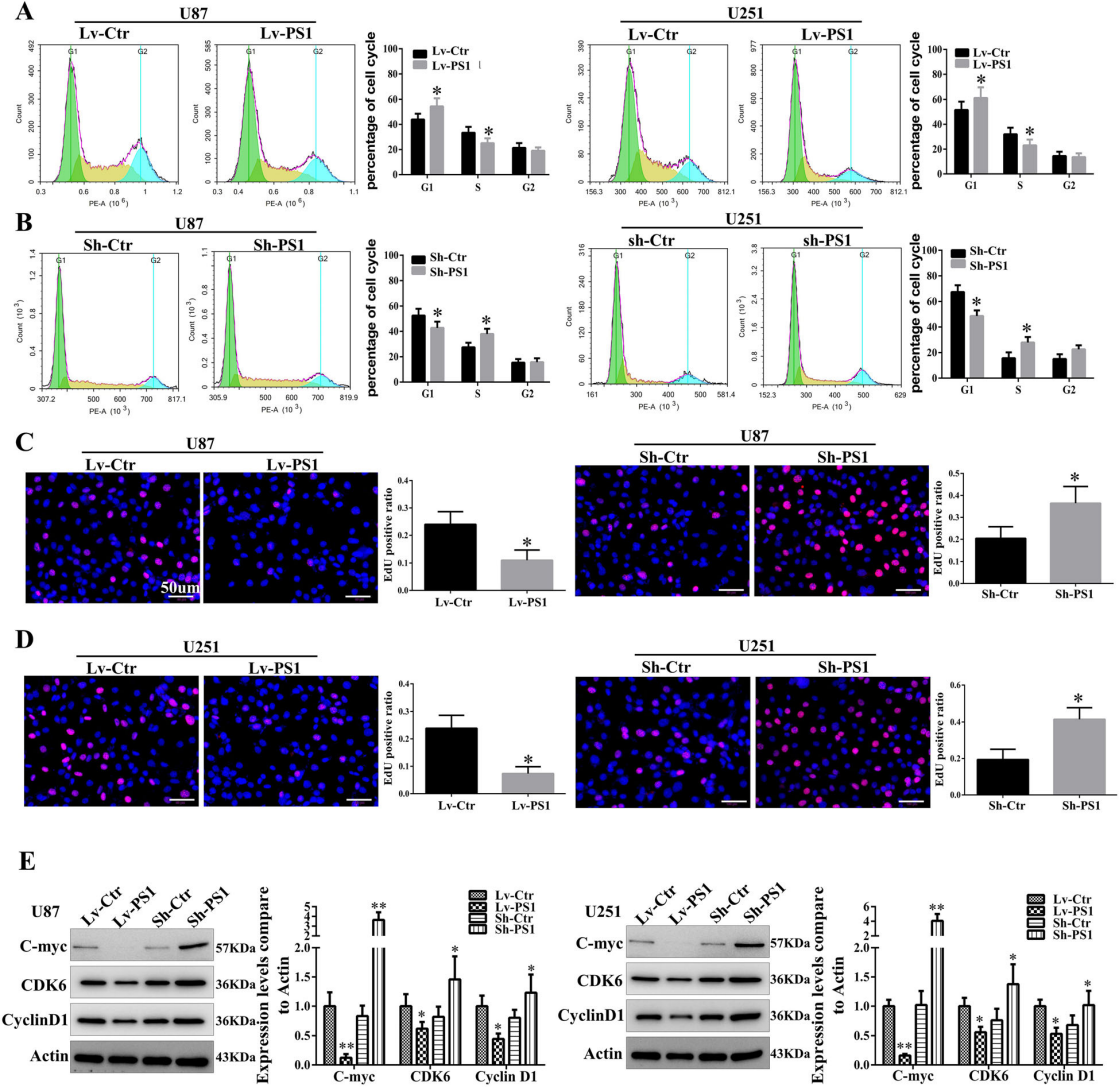
**a** U87 and U251 cells were over-expressed of Presenilin1, then were used to flow cytometry assays to analyse the cell cycle. Histograms showing the percent of cell at G1, S and G2 phase. **b** Flow cytometry assays were applied to investigate the influence of down expression of Presenilin1 on cell cycle in U87 and U251 cells. **c** Edu staining to show the proliferation states of U87 and U251 cells after Presenilin1 were up-regulated, histograms showing the Edu positive cell ratio in each groups, Scale bars = 50um. **d** Representative Edu images and histograms to show the influence of Presenilin1 repression on glioma proliferation, Scale bars = 50um. **e** Western-blot assays to show the expression of C-myc, CDK6 and CyclinD1 when Presenilin1 down- or up-regulation in U87and U251 cell. * *p* < 0.05,** *p* < 0.01

We observed that Presenilin1 exerted a negative effect on glioblastoma cell growth. To further investigate the underlying mechanism, flow cytometry assays were performed to analyze the changes in the cell cycle after the loss or gain of function of Presenilin1. The results revealed that the overexpression of Presenilin1 arrested the cell cycle at the G1/S phase but did not alter the G2 phase in the U87 and U251 cell lines (Fig. 3a). In contrast, the downregulation of Presenilin1 significantly promoted the cell cycle from the G1 phase to the S phase (Fig. 3b). Additionally, Edu staining showed that the stimulation of Presenilin1 clearly decreased the positive rates of Edu in U87 and U251 cells (Fig. 3c). However, blocking Presenilin1 significantly elevated the positive rate of Edu in U87 and U251 cells (Fig. 3d). Next, we detected the molecules that regulate the G1/S cell cycle and found that the expression of C-myc, CDK6 and cyclin D1 were distinctly decreased with Presenilin1 stimulation. In contrast, the above cell cycle regulators were upregulated after Presenilin1 repression (Fig. 3e). These results revealed that Presenilin1 negatively mediated the progression of the G1/S phase of the cell cycle by repressing the expression of C-myc, CDK6 and cyclin D1.

### Presenilin1 negatively regulates Wnt/β-catenin signaling by promoting β-catenin phosphorylation and degradation in glioblastoma

**Fig. 4:**
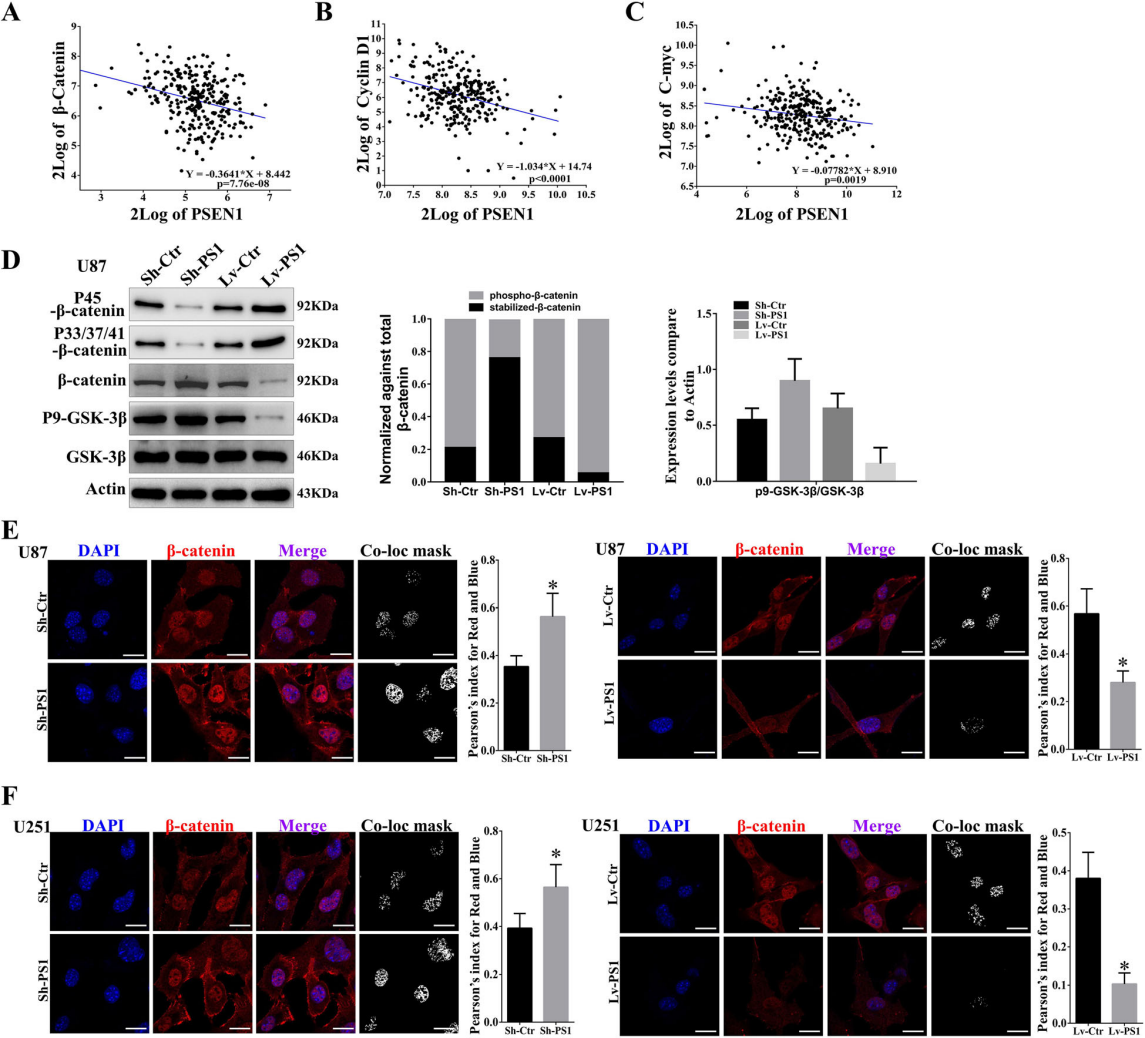
**a-c** the correlation of Presenilin1 with β-catenin, CyclinD1 and C-myc from French-284-glioma dataset. **d** Western-blot assays to investigate the expression levels of p45-β-catenin, p33/37/41-β-catenin, total β-catenin, p9-GSK-3β and GSK-3β in U87 cells after down- or up-expression of Presenilin1. **e-f** The expression of β-catenin was detected by IF assays in U87 and U251 cells after down- or up-expression of Presenilin1. the co-localization mask and Pearson’s index were analyzed by Image J. scale bar = 20um, * *p* < 0.05,** *p* < 0.01

Increasing evidence indicates that Wnt/β-catenin signaling promotes cancer progression and that Presenilin1 could promote β-catenin degradation in embryonic stem cells. To test whether Presenilin1 mediates the Wnt pathway in glioblastoma, we analyzed the correlation between Presenilin1 and Wnt-related molecules. We found that β-catenin mRNA levels were significantly and negatively correlated with Presenilin1 mRNA levels in glioma patients (Fig. 4a). Additionally, the Wnt target genes C-myc and cyclin D1 were also negatively correlated with Presenilin1 mRNA levels in glioma patients (Fig. 4b & c). Next, we detected the phosphorylation of β-catenin and GSK-3β in U87 and U251 cells. The results showed that the Ser45 site and Thr41/Ser37/Ser33 site phosphorylation levels of β-catenin were significantly decreased with Presenilin1 downregulation and increased with Presenilin1 overexpression. Conversely, the stabilized β-catenin level was elevated when Presenilin1 was downregulated and reduced when Presenilin1 was upregulated in U87 cells, indicating that Presenilin1 decreased β-catenin stabilization by promoting degradation-related phosphorylation at Ser45 and Thr41/Ser37/Ser33. Additionally, we observed that the phosphorylation of GSK-3β was negatively correlated with the expression of Presenilin1, suggesting Presenilin1 as an activator of GSK-3β (Fig. 4d and Additional file 1: Figure S1C). Considering that GSK-3β was the main promoter in phosphorylating β-catenin at the Thr41, Ser37, and Ser33 sites, we concluded that Presenilin1 inhibited Wnt/β-catenin signaling by directly phosphorylating β-catenin at Ser45 and activated GSK-3β to indirectly phosphorylate β-catenin at the Thr41, Ser37, and Ser33 sites. Next, we utilized immunofluorescence (IF) to show the effect of Presenilin1 on the distribution of β-catenin. As shown in Fig. 4e, β-catenin was primarily localized in the nucleus of U87 cells, and Presenilin1 deficiency distinctly elevated the expression of β-catenin. Additionally, we analyzed the colocalization of β-catenin and DAPI and found that the Pearson index was significantly increased after Presenilin1 repression. In contrast, we observed that the activation of Presenilin1 clearly decreased the expression of β-catenin and the colocalization of β-catenin to DAPI, suggesting that the repression of Presenilin1 maintained β-catenin stabilization and transported it to the nucleus. We repeated IF assays in U251 cells and obtained similar results (Fig. 4f). The above results illustrated that Presenilin1 exerted an inhibitory effect on the Wnt/β-catenin pathway by promoting the phosphorylation of β-catenin, which could completely explain the antiproliferative role of Presenilin1 in GBM.

### Presenilin1 regulates glioblastoma proliferation in vivo

**Fig. 5:**
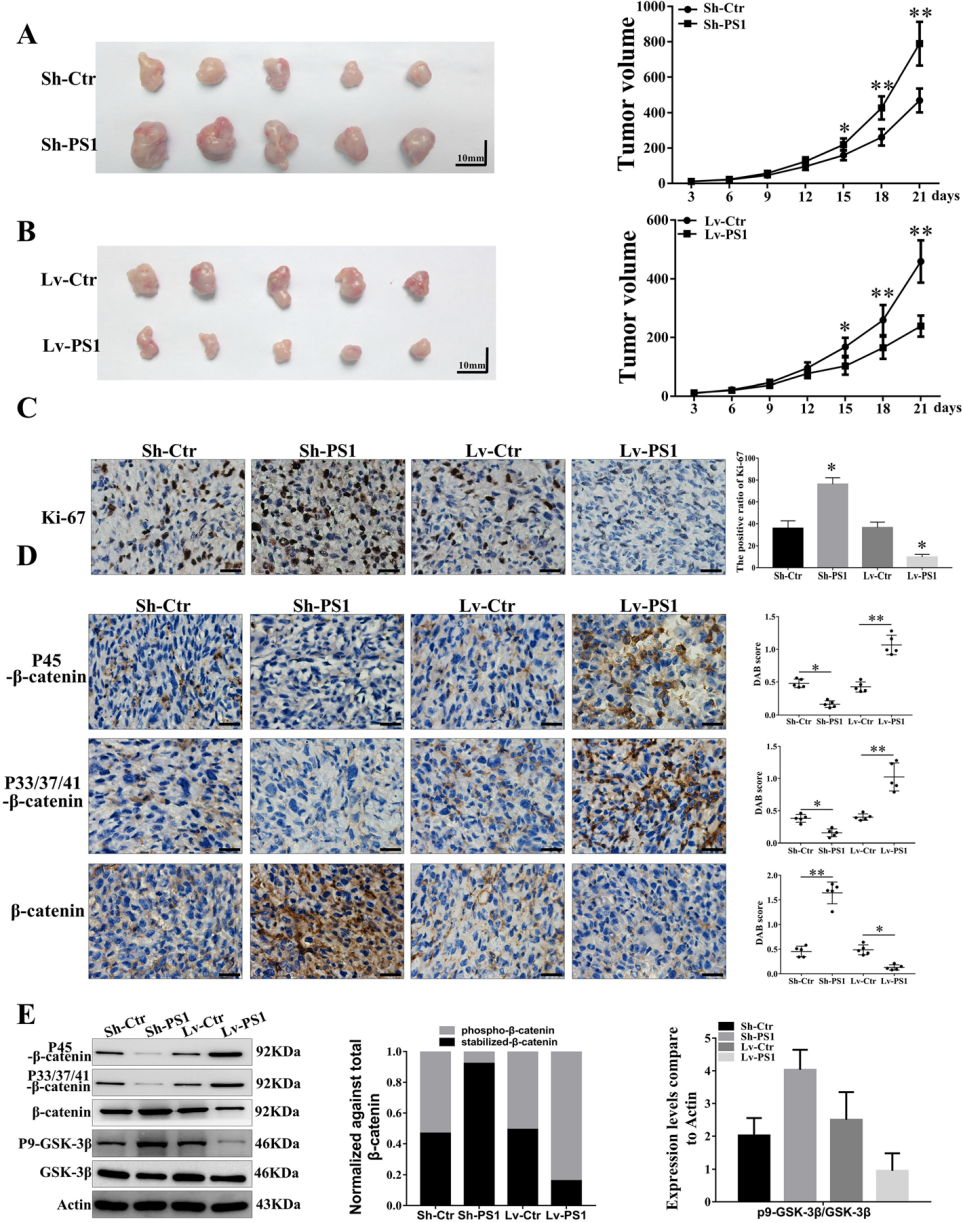
**a-b** Subcutaneous transplanting gliomas were utilized to analyse the function of Presenilin1 on glioma in vivo, tumor values were measured every-3 days and calculated to histogram. **c** Representative IHC images of Ki67 expression in 4 groups of subcutaneous glioma tissues, scale bar = 50um. **d** The expression of p45-β-catenin, p33/37/41-β-catenin and total β-catenin in 4 groups of subcutaneous glioma tissues were detected by IHC, scale bar = 50um. * *p* < 0.05,** *p* < 0.01. **e** Western-blot assays to investigate the expression levels of p45-β-catenin, p33/37/41-β-catenin, total β-catenin, p9-GSK-3β and GSK-3β in U87 cells after down- or up-expression of PSEN1

To further verify the role of Presenilin1 in GBM, we first established a transplanting GBM model with U87-Sh-PS1 cells, U87-Lv-PS1 cells and control cells. We found that the knockout of Presenilin1 significantly promoted the growth of subcutaneous GBM compared to the control (Fig. 5a). In contrast, Presenilin1 overexpression clearly alleviated the growth of GBM compared to the control (Fig. 5b). The IHC staining of Ki-67 to show the proliferation states of subcutaneous GBM tissues showed that the positive rate of Ki-67 in the sh-PS1 groups was significantly higher than that in the control groups. In contrast, the Ki-67-positive rates in the Lv-PS1 groups were clearly lower than those in the control groups (Fig. 5c). Western blot and IHC assays indicated that the direct and indirect phosphorylation levels of β-catenin were significantly elevated in the Lv-PS1 groups and reduced in the Sh-PS1 groups. Stabilized β-catenin was distinctly decreased in the Lv-PS1 groups and increased in the Sh-PS1 groups (Fig. 5d & e). These results verified that Presenilin1 exerted a repressive function in GBM via the Wnt/β-catenin pathway in vivo.

### Overexpression of Presenilin1 represses the growth of intracranial glioblastoma

**Fig. 6:**
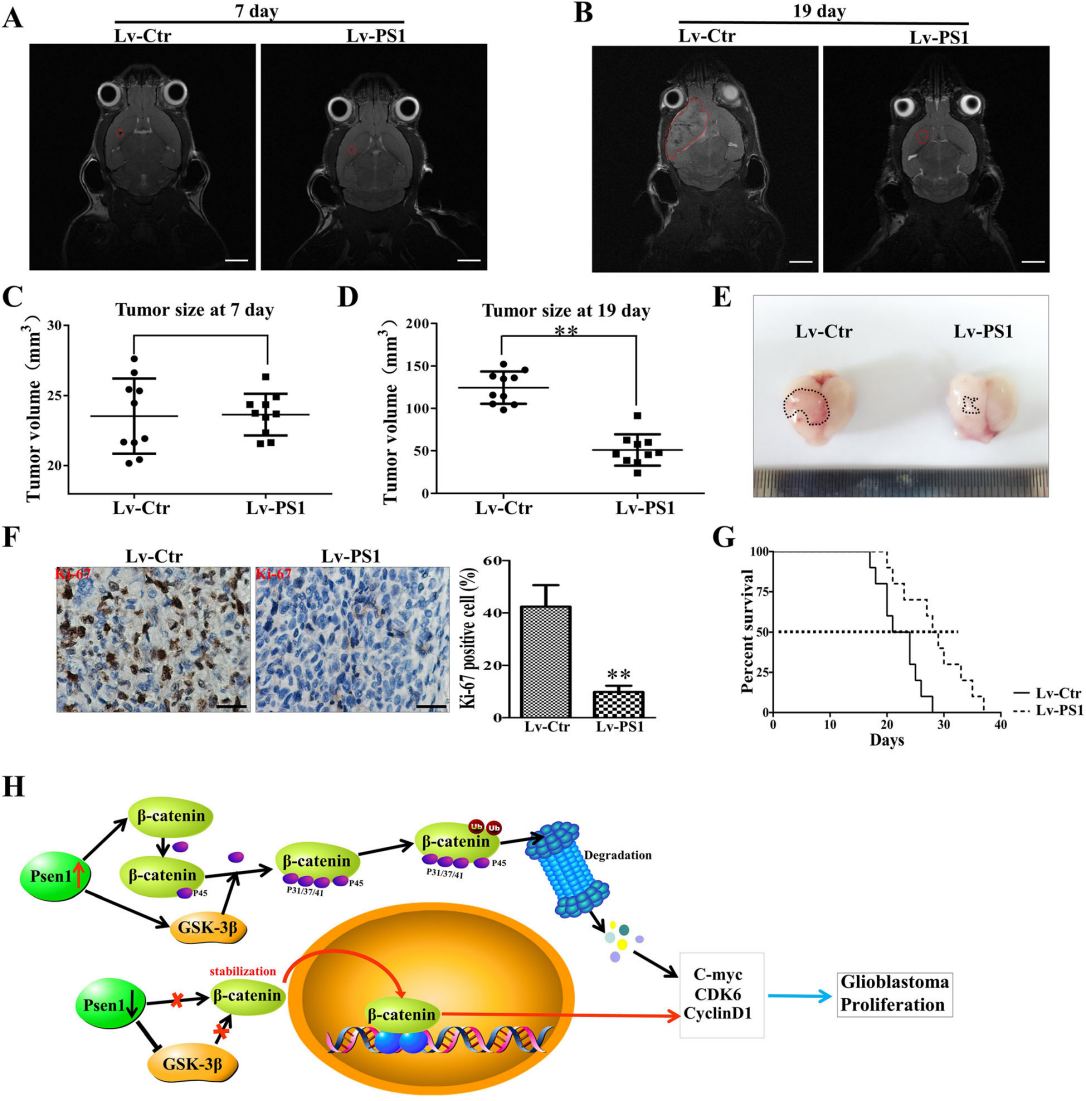
**a-d** Intracranial xenografts of gliomas were established with GL2161 cells after up-regulation of Presenilin1, representative MRI image showing the the tumor size at 7 days and 19 days after transplating in each groups, scale bar = 5 mm. Histograms showing the tumor values at 7 days and 19 days after transplating in each groups. **e** The representative gross image of glioma after dissected from mice in each group. **f** IHC staining of Ki-67 to show the proliferation state of transplating glioma, Scale bar = 50um. **g** The survival curve of glioma-bearing mice was analyzed by Kaplan-Meier. **h** The schematic model: Presenilin1 exert anti-glioma function by increasing the phosphorylation of β-catenin and degradation, then arrest cell cycle at G1/S phase.* *p* < 0.05,** *p* < 0.01

We transplanted U87-Lv-PS1 cells and control cells to the right striatum of nude mice and found that the upregulation of Presenilin1 sharply repressed the growth of intracranial glioma by MRI detection (Fig. 6a-b). The tumor value in the Lv-PS1 group was significantly smaller than that in the control group on day 19 after transplantation (Fig. 6c-d). Likewise, gross observation also demonstrated that the upregulation of Presenilin1 slowed the growth of orthotopic glioblastoma (Fig. 6e). In addition, the proliferative state was alleviated after Presenilin1 upregulation by detecting Ki-67 (Fig. 6f). Importantly, the upregulation of Presenilin1 clearly prolonged the survival time of glioblastoma-bearing mice (Fig. 6g). Based on the in vitro and in vivo results, we confirmed that Presenilin1 exerted an anti-growth function by increasing the phosphorylation and decreasing the stabilization of β-catenin, then arresting the cell cycle at the G1/S phase, eventually repressing the cell proliferation of glioblastoma (Fig. 6h).

## Discussion

The aim of the present study was to explore the expression profile and functions of Presenilin1 in glioma. Our results indicated that Presenilin1 was downregulated in glioma and positively correlated with the prognosis of glioma and GBM patients. We found that the level of Presenilin1 was negatively correlated with GBM proliferation. Furthermore, Presenilin1 repressed GBM growth by arresting the cell cycle at the G1/S phase. Additional results demonstrated that Presenilin1 regulates the cell cycle by altering CDK6, C-myc and Cyclin D1 in GBM cells. Finally, we confirmed that β-catenin phosphorylation and degradation were involved in Presenilin1-suppressed GBM proliferation in vitro and in vivo. Overall, our data also suggest that the overexpression of Presenilin1 might be a promising agent for human GBM.

We observed that the low expression levels of Presenilin1 indicated a worse prognosis in glioma patients. The acceleration of GBM cell growth is well known to be associated with cancer development and causes a dismal prognosis [15]. Our study showed that the downregulation of Presenilin1 in GBM cells led to a significant increase in growth in human GBM cells (U87 and U251). The upregulation of Presenilin1 had the opposite effect. Cancer is characterized by uncontrolled proliferation resulting from the aberrant activity of various cell cycle proteins [16]. Our data demonstrate that Presenilin1 may suppress GBM cell growth by arresting the cell cycle at the G1/S phase by downregulating the expression of CDK6, C-myc and cyclin D1, and Presenilin1 overexpression had the opposite effect. The elevation of those cyclins is considered a hallmark for GBM proliferation and has been associated with poor prognosis in GBM patients [17–19]. In this study, we found that Presenilin1 can negatively regulate GBM cell proliferation, which could explain the negative correlation between Presenilin1 and GBM patient prognosis.

Until now, a number of studies have investigated the bidirectional function of Presenilin1 in cancer. On one hand, Ham, S et al. reported the inverse correlation of AD and cancer observed in the Presenilin1-KO mouse model of AD [20]. The downregulation of Presenilin1 in hepatocellular carcinoma cells promotes cell survival and decreases cell apoptosis to modify radioresistance and chemotherapeutic tolerance [14]. Similarly, the expression level of Presenilin1 is negatively correlated with bladder cancer chemoresistance [12]. The repression of Presenilin1 suppresses cell apoptosis, and the activation of Presenilin1 enhances cell death in bladder cancer. Downregulating Presenilin1 contributes to the radiation and chemotherapy resistance of esophageal carcinoma [21]. Additionally, Significant negative associations between Presenilin1 rs165934 genes and OS have been found in Chinese epithelial ovarian cancer (EOC) patients [10]. On the other hand, Presenilin1 is significantly upregulated in gastric cancer and positively correlated with lymph node metastasis and poor OS. Presenilin1 cleaves E-cadherin and the β-catenin complex, releasing β-catenin to translocate into the nucleus and activate target genes [11]. Here, we report the antitumor role of Presenilin1 in glioblastoma by repressing cell proliferation, exploring the role of Presenilin1 in cancer and connecting this AD-protein to glioblastoma.

In this study, we report that the downregulation of Presenilin1 enhanced glioblastoma growth in vitro and in vivo. Previously, Daniel P et al. reported that orthotopically implanted glioma is significantly reduced in Tg PS1/APPsw mice compared to their wild-type littermate, suggesting that AD does not constitute a favorable environment to support glioma growth [22]. However, the tumor volume of Tg PS1/APPsw mice was slightly larger (no significant difference) than that of Tg APPsw mice, implying that PS1 dysregulation could reverse the depression of glioma growth when APP is overproduced. In keeping with the above observations, our results support Presenilin1 as a tumor inhibitor by repressing the β-catenin signal. In addition to uncontrollable tumor cell proliferation, GBM is also characterized by an extensive aggressive nature, which enhances tumor cell infiltration into the surrounding brain parenchyma and leads to inevitable recurrence even after gross total resection [23]. Additionally, the elevated vascularity and tumor necrosis of GBM make these tumors prone to recurrence with an extremely dismal outlook [24]. Therefore, whether Presenilin1 is also involved in these pathological processes should be explored in the future.

Presenilin1 is the main substrate of γ-secretase, which is well studied in the processing of the amyloid-β protein precursor. The constituents of the γ-secretase complex are four transmembrane proteins: heterodimeric presenilins (either Presenilin1 or Presenilin2), NCT, Aph-1, and Pen-2 [25]. Currently, as the main active component of γ-secretase, Presenilin1 is identified to participate in the cleavage of type I membrane receptors such as Notch and P75^NTR^. The cleavage of Notch receptors to release their intracellular domain (NICD) is vital for signal transduction [26]. The NICD is sorted into the cytosol and is a well-known transcription factor for several genes mostly involved in cell survival and differentiation [27]. In glioma stem cells (GSCs), Notch signaling is highly activated and contributes to maintaining stem cell-like properties [28]. The γ-secretase inhibitor prevents the release of the active NICD from the receptor and results in repression of proliferation and neurosphere formation, as well as promoting apoptosis in GBM cells [29, 30]. Additionally, P75^NTR^ is upregulated in GBM and mediates the function of pro-BDNF to inhibit glioma cell growth [31]. Thus, Presenilin1 may cleave P75^NTR^ to relieve the pro-growth function of pro-BDNF. Here, we found that Presenilin1 repressed GBM by increasing the degradation-dependent phosphorylation of β-catenin at the 45 site and 33/37/41 sites. Our previous study demonstrated that sortilin suppressed the GSK-3β/β-catenin pathway in GBM cells [32]. Moreover, sortilin, as a single transmembrane domain type I receptor, could be cleaved to result in the shedding of the luminal domain [33]. Thus, we suspect that Presenilin1 may represses WNT/β-catenin signaling via its participation in sortilin cleavage, which will be investigated in future studies.

## Conclusions

In summary, our results suggest that Presenilin1 is frequently downregulated in human glioma tissues and acts as an antitumor molecule to repress cell proliferation during the progression of glioblastoma in vitro and in vivo. Mechanistically, Presenilin1 regulates Wnt/β-catenin signaling by promoting the phosphorylation process of β-catenin, leading to repressed CDK6, Cyclin D1 and C-myc and hindering cell cycle progression by arresting the G1/S checkpoint. Therefore, we first report that the AD-related protein Presenilin1 plays an antitumor role in glioblastoma and offer a novel target for the diagnosis and treatment of glioblastoma patients.

## Supplementary information

**Additional file 1: Figure S1**. **A:** Kaplan-Meier analysis of overall survival rates for low grade glioma patients. **B:** The knockdown effect and overexpression effect of lentivirus in GL261 cells was detected by western-blot assays. **C:** Western-blot assays to investigate the expression levels of p45-β-catenin, p33/37/41-β-catenin, total β-catenin, p9-GSK-3β and GSK-3β in U251 cells after down- or up-expression of PSEN1.

## Data Availability

The data used or analyzed during this study are included in this article and available from the open access website or platform.

## Abbreviations

BDNF: Brain Derived Neurotrophic Factor
CDK6: Cyclin dependent kinase 6
CK-1α: Casein Kinase-1α
GSK-3β: Glycogen synthase kinase-3β
P75^NTR^: P75 Neurotrophic factor receptor
