# Supplementary figures and images for: Presenilin1 exerts antiproliferative effects by repressing the Wnt/β-catenin pathway in glioblastoma
Source: Cell Commun Signal. 2020 Feb 11;18:22. doi: 10.1186/s12964-019-0501-9 (PMC7014622; doi:10.1186/s12964-019-0501-9)

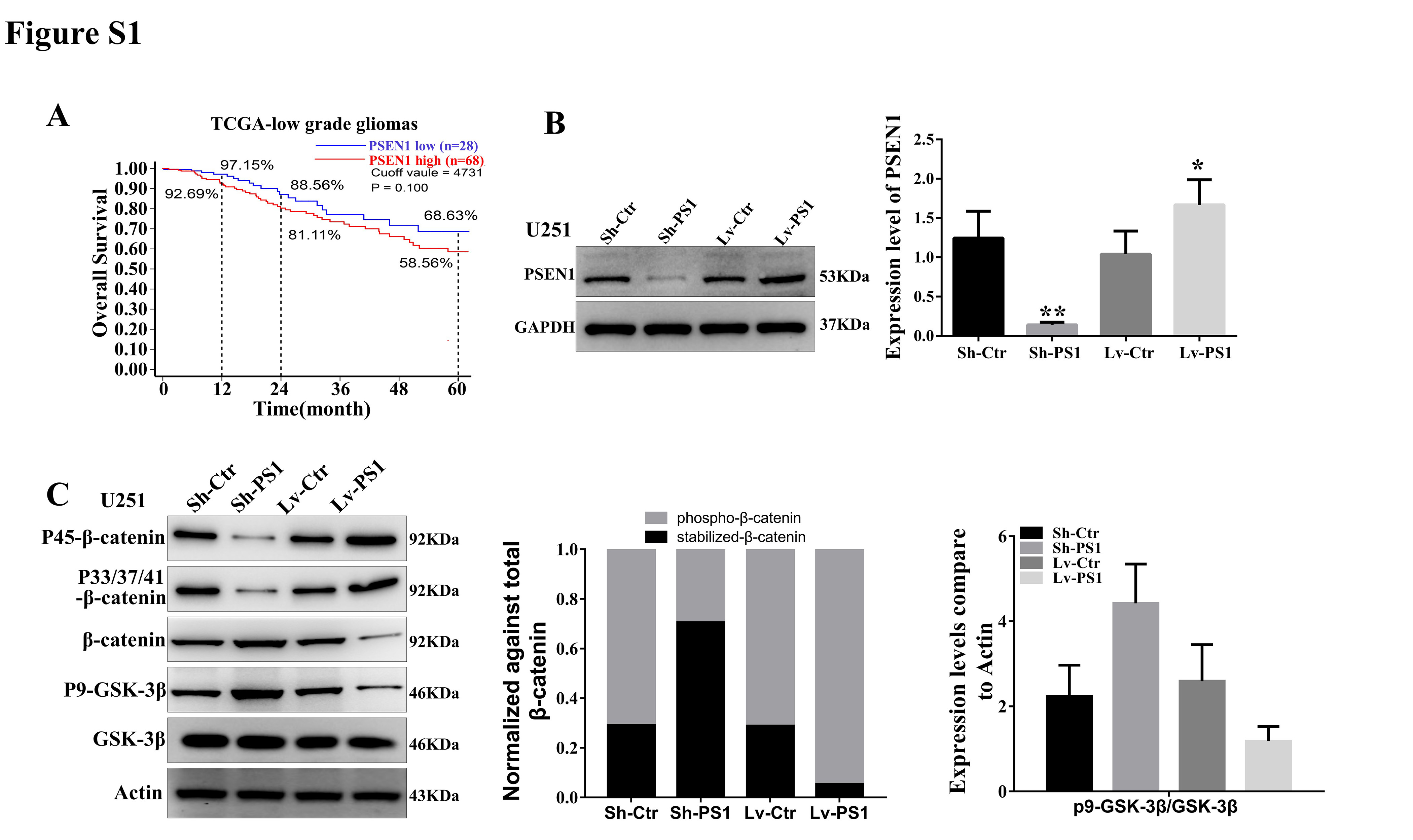

Supplement: Supplementary file 2 — Additional file 1: Figure S1. A: Kaplan-Meier analysis of overall survival rates for low grade glioma patients. B: The knockdown effect and overexpression effect of lentivirus in GL261 cells was detected by western-blot assays. C: Western-blot assays to investigate the expression levels of p45-β-catenin, p33/37/41-β-catenin, total β-catenin, p9-GSK-3β and GSK-3β in U251 cells after down- or up-expression of PSEN1. [file 12964_2019_501_MOESM2_ESM.jpg]
